# Memory and betweenness preference in temporal networks induced from time series

**DOI:** 10.1038/srep41951

**Published:** 2017-02-03

**Authors:** Tongfeng Weng, Jie Zhang, Michael Small, Rui Zheng, Pan Hui

**Affiliations:** 1 HKUST-DT System and Media Laboratory, Hong Kong University of Science and Technology, HongKong; 2 Centre for Computational Systems Biology, Fudan University, Shanghai, China; 3 The University of Western Australia, Crawley, WA 6009, Australia; 4 Mineral Resources, CSIRO, Kensington, WA, Australia

## Abstract

We construct temporal networks from time series via unfolding the temporal information into an additional topological dimension of the networks. Thus, we are able to introduce memory entropy analysis to unravel the memory effect within the considered signal. We find distinct patterns in the entropy growth rate of the aggregate network at different memory scales for time series with different dynamics ranging from white noise, 1/f noise, autoregressive process, periodic to chaotic dynamics. Interestingly, for a chaotic time series, an exponential scaling emerges in the memory entropy analysis. We demonstrate that the memory exponent can successfully characterize bifurcation phenomenon, and differentiate the human cardiac system in healthy and pathological states. Moreover, we show that the betweenness preference analysis of these temporal networks can further characterize dynamical systems and separate distinct electrocardiogram recordings. Our work explores the memory effect and betweenness preference in temporal networks constructed from time series data, providing a new perspective to understand the underlying dynamical systems.

Characterizing and unveiling evolutionary mechanisms from experimental time series is a fundamental problem which has attracted continuous interest over several decades. Going beyond conventional nonlinear techniques such as Lyapunov exponent^1^, symbolic dynamics approaches^2^,^3^, and surrogate methods^4^,^5^, intensive attention has focused on understanding dynamics of time series from the complex network perspective^6^,^7^,^8^,^9^,^10^,^11^,^12^,^13^,^14^. Depending on the definition of nodes and links, the previous transformation methods can be broadly classified into four categories: proximity networks^6^,^7^,^8^,^9^, visibility graphs^10^, recurrence networks^11^,^12^,^13^, and transition networks^14^. Intensive studies demonstrate that complex network measures are shown to provide an effective tool at characterizing dynamics^6^, identifying invariant substructures^7^,^8^,^9^,^10^, and describing attractor structure organization^11^,^12^,^13^,^14^. In this sense, network science offers an alternative perspective to characterize dynamical properties of experimental time series.

Generally, previous studies have mainly focused on a static network representation of time series. Amongst the static network representations, there is a growing industry in characterizing and exploring the time-varying nature of dynamical system^15^,^16^,^17^,^18^. Nonetheless, for describing adaptive systems, a desirable way is to use temporal networks instead of static ones^19^,^20^,^21^,^22^. In fact, it has been shown that the temporal network perspective via features such as accessibility behavior^22^, betweenness preference phenomenon^23^ and causality-driven characteristics^24^, can help us to better understand the dynamical variation of real systems deviating significantly from what one would expect from static network models. In particular, causality-driven characteristics provide an important advance and allow us to uncover the effect of low-order memory on temporal networks for exploring diffusion behaviors^25^. More significantly, the memory effect plays a key role for accurately understanding real systems ranging from traffic prediction^25^ and epidemics spreading^26^ to information search^27^.

In this paper, we propose a methodology for transforming time series into temporal networks by encoding temporal information into an additional topological dimension of the graph, which describes the “lifetime” of edges. We then introduce the memory entropy technique to reveal the memory effect within different types of time series including: white noise; 1/f noise; autoregressive (AR) process; and, periodic to chaotic dynamics. We find that time series with different underlying dynamics exhibit distinct memory effect phenomena which in turn can be used to characterize and classify the underlying dynamics. Interestingly, an exponential scaling behavior exists for a chaotic signal and the memory exponent is consistent with the largest Lyapunov exponent. We show that the memory exponent is capable of detecting and characterizing bifurcation phenomena. Application to human electrocardiogram (ECG) data during sinus rhythm (SR), ventricular fibrillation (VF), and ventricular tachycardia (VT) shows that such a memory exponent can accurately characterize and classify the healthy and pathological state of the heart. Moreover, we find that the betweenness preference analysis can further explore the essential difference among distinct chaotic systems and differentiate the human cardiac system under distinct states (i.e., SR, VF, and VT).

## Results

### From time series to temporal networks

**Figure 1:**
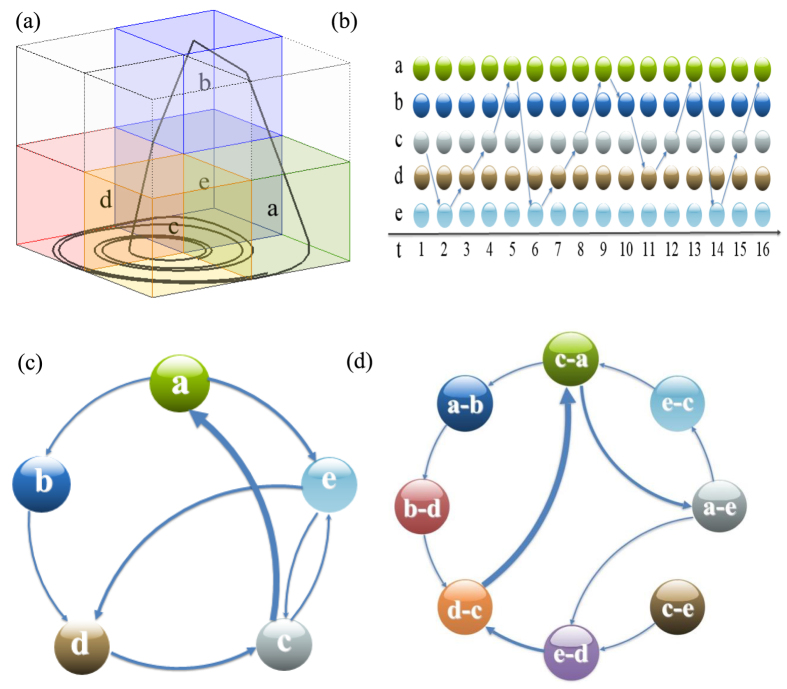
Schematic diagram depicting the process of transforming a time series into a temporal network. (**a**) Partition the phase space into 8 hyper-cubers. The trajectory flow thereby creates time-stamped edges in (**b**) the associated temporal network *G*^*T*^. (**c**) The null aggregate network *G*^(0)^ and (**d**) the one-step memory network *G*^(1)^ constructed from *G*^*T*^. The line width represents the number of transition times between two nodes.

We start from the construction of the temporal network from a dynamical system. Let 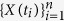 be a trajectory flow Γ of a dynamical system in *m*-dimensional phase space. We first partition the whole space into *N* non-overlapping cells with an equivalent size. For instance, the phase space shown in Fig. 1(a) is partitioned into 8 cells. By considering each cell as a node of the network, a temporal network representation *G*^*T*^ of a given time series is achieved as follows: we denote a link between two nodes as being “active” with a particular “lifetime”. Specially, a time-stamped edge (*υ, ω*; *t*) between nodes *υ* and *ω* exists whenever the trajectory flow Γ performs a transition from cell *υ* to cell *ω* at time stamp *t*. As illustrated in Fig. 1(b), the time-stamped edge (*c, e*; 1) means that the trajectory flow Γ hops from cell *c* to cell *e* at time stamp *t* = 1. Similarly, the trajectory flow travels from cell *e* to cell *d* at time stamp *t* = 2 represented by the time-stamped edge (*e, d*; 2). This new definition of links is distinct from that of previous static network representations in which edges are established during the whole time window^6^,^7^,^8^,^9^,^10^,^11^,^12^,^13^,^14^. Specifically, for the previous static network representations, a time-stamped edge (*υ, ω*; *t*) exists for all time stamps *t*, while the time-stamped edge (*υ, ω*; *t*) occurs at some time stamps *t* in a temporal network representation. Based on time-stamped edges (*υ, ω*; *t*) ∈ *E*, we construct a temporal network from time series by unfolding temporal information *t* into an additional topological dimension. This configuration obviously ensures the one-to-one mapping between a time series and a temporal network. Hence, the temporal network will typically represents a unique time series. Note that in the process of constructing temporal networks from time series, the first step is to segment phase space into several cells. Of course, an ideal partition is to make each cell infinitesimal. However, such a partition is usually impractical due to the finite length of an observed time series. Instead, our objective is to ensure that distinct states of an attractor are distinguishable and similar states are grouped into the same cell. A specific example of this type of partitions can be referred to the ordinal partition method^28^.

### The memory entropy analysis of different dynamics

**Figure 2:**
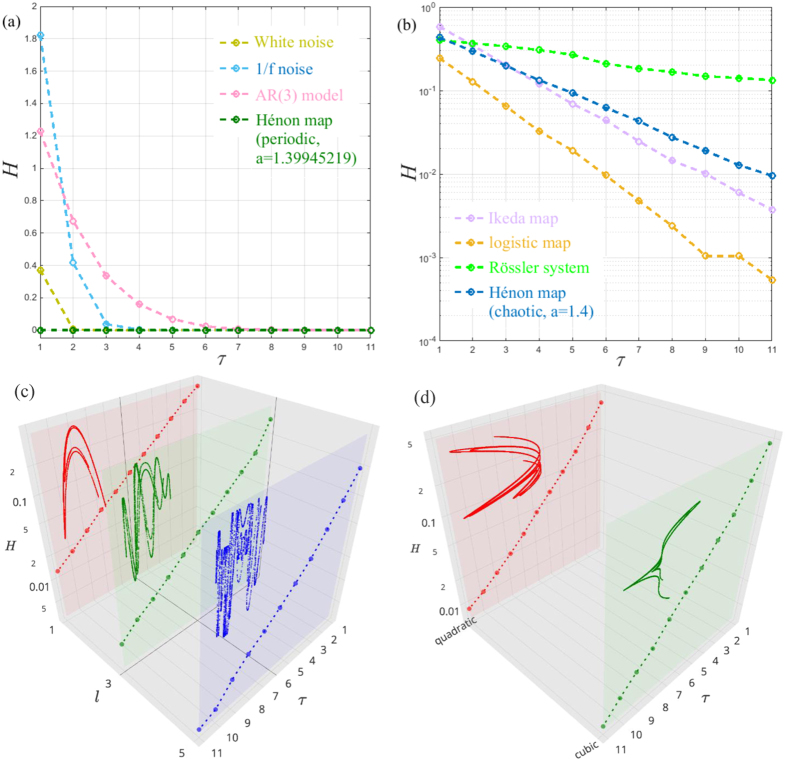
The entropy growth rate *H* of a *τ*-order aggregate network as a function of the order *τ* for (**a**) White noise, 1/f noise, AR(3) model *s*_*n*_ = 0.8*s*_*n*−1_ − 0.5*s*_*n*−2_ + 0.7*s*_*n*−3_ + *ε*_*n*_ (*ε*_*n*_ is generated from white noise), and the Hénon map: 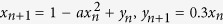 and (**b**) Chaotic time series (i.e., the Hénon map, the Ikeda Map: *x*_*n*+1_ = 1 + 0.9 (*x*_*n*_cos*t*_*n*_ − *y*_*n*_sin*t*_*n*_), *y*_*n*+1_ = 0.9 (*x*_*n*_sin*t*_*n*_ − *y*_*n*_cos*t*_*n*_), where 
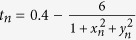
, the logistic map: *x*_*n*+1_ = 4*x*_*n*_(1 − *x*_*n*_), and the Rössler system: 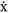 = −(y + z), 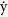 = x + 0.2y, 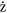 = 0.2 + z(x − 5.7)). Note that here we choose the partition *N* = 2500, 1600, 900, 100 for the Hénon map, the Ikeda Map, the logistic map, and the Rössler system, respectively. The entropy growth rate *H* as a function of the order *τ* for the Hénon map in chaotic regime for (**c**) different time delays *l* and for (**d**) quadratic and cubic nonlinear transformations of the phase space. The planar insets show the corresponding phase portraits.

After constructing the temporal network *G*^*T*^ from a given time series, we build a null aggregate network *G*^(0)^ in which a directed edge (*υ, ω*) from node *υ* to *ω* exists whenever a time-stamped edge (*υ, ω*; *t*) emerges in *G*^*T*^ for some *t* and the weight *w(υ, ω*) measures how many times the directed edge (*υ, ω*) occurs in the temporal network *G*^*T*^ (see Fig. 1(c)). From the null aggregate network *G*^(0)^, we construct the consecutive memory network *G*^(*τ*)^ = (*V*^(*τ*)^, *E*^(*τ*)^), determined by the memory factor *τ*. Specifically, each node 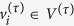 represents a possible *τ*-step paths in the network *G*^(0)^ such that 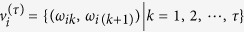, where 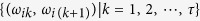 is a set of consecutive edges in *G*^(0)^. Here, the consecutive edges we mean the consecutive paths under the constraint of the edge direction with respect to the structure organization of *G*^(0)^. Edges *E*^(*τ*)^ in *G*^(*τ*)^ are defined by all possible paths of length 2*τ* + 1 in *G*^(0)^. For example, we construct a one-step memory network *G*^(1)^ from the previous temporal network *G*^*T*^ as illustrated in Fig. 1(d). We then apply the memory entropy (ME) approach (see methods) on the consecutive memory network *G*^(*τ*)^ to reveal the effects of memory on shaping dynamics. Here, we investigate the transformed networks constructed from different types of time series, including white noise, 1/f noise, AR model, periodic signals, and chaotic signals. For each prototypical system, the length of the time series is *n* = 2 × 10^4^ (after neglecting the first 4000 points). We find that temporal networks generated from different types of time series show distinct memory behaviors (see Fig. 2(a) and (b)). Specially, for white noise and 1/f noise, the entropy growth rate decreases sharply to zero in less than two or three memory scales, respectively, while it monotonically decreases on finite memory scales (i.e., *τ* ≤ 6) and then stabilizes to zero for the AR(3) model. Similarly, for a periodic signal (the Hénon map in a periodic regime for example) the entropy growth rate remains constant for all memory scales. In comparison, the entropy growth rates of chaotic signals, such as the logistic map, the Ikeda map, the Rössler system, and the Hénon map, monotonically decrease over the whole memory scale window. We call this behavior the memory effect on the time series. The observation shows that the memory effect plays an important role in shaping dynamical systems, comparable to the importance of the memory effect on real systems reported in ref. 25.

These findings can be explained when referring to the evolution processes of these distinct systems. Specially, for white noise that is derived from a random process, a weakly one-step memory phenomenon exists due to the combined effect of finite data length and the coarse-grained process in the temporal network transformation. Unlike white noise, 1/f noise contains temporal connections which consequently results in the two-step memory behavior. Similarly, for the AR(3) model, the trajectory state at time *t* thoroughly depends on the previous three state information. Thereby, it shows memory characteristics over more time steps, compared with that of white noise and 1/f noise. In contrast, a periodic trajectory presents a clearly repeated behavior in phase space. Then, there is no memory effect on its trajectory that shows a constant value of the entropy growth rate. However, for a chaotic attractor, the skeleton is made up of an infinite number of unstable periodic orbits (UPOs)^29^. The trajectory evolution of a chaotic system will typically hop among these UPOs resulting in a dramatically distinct behavior of the entropy growth rate versus the memory scale.

Remarkably, for a chaotic system, an apparently exponential behavior emerges between *H(τ*) and *τ* (i.e., *H(τ*) ∝ exp(−*ρτ*)) as illustrated in Fig. 2(b). We thus define *ρ* as the memory exponent of a dynamical system. Such interesting scaling behavior can be anticipated by the encounter probability Φ(*k*) for an orbit of period *k* in a chaotic system. It is known that the trajectory usually approaches a UPO for a certain time interval until it is captured by the stable manifold of another UPO and so on. For any periodic orbit of period *k*, the main dependence of Φ(*k*) on *k* can be approximated by refs 30 and 31


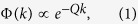


where *Q* = *λ* + *m*_1_ − *m*_0_, *λ, m*_1_ and *m*_0_ are the largest Lyapunov exponent, the metric entropy and the topological entropy of the chaotic attractor, respectively. For each memory scale *τ*, the memory effect is based on the behaviors of *τ*-step consecutive paths in the null aggregate network *G*^(0)^, which is largely dominated by the encounter probability Φ(*k*) in phase space. Thus, the memory effect on a chaotic time series demonstrates the same behavior as described in Eq. (1). Moreover, we find that the memory exponents for the Rössler system, the logistic map, the Ikeda map, and the Hénon map are 0.09, 0.658, 0.509, and 0.402, respectively. They are in good agreement with the largest Lyapunov exponents *λ* as reported in refs 1 and 32. Empirical findings suggest that the memory exponent *ρ* presents a strong connection with the largest Lyapunov exponent *λ* for chaotic time series. In fact, both of them are quantities that characterize the trajectory evolution of dynamical systems. For the chaotic system with a larger *λ*, infinitesimally close trajectories will be separated more rapidly. In this situation, the underlying connection of close trajectories will be lost more quickly. Such a characteristic is captured by the memory effect analysis, where the entropy growth rate *H(τ*) consequently decreases more rapidly, which in turn results in a higher memory exponent *ρ* and vice versa.

Here, we note that since the random processes (i.e., white noise and 1/f noise) have no attractor structure, we derive the temporal networks from their scalar time series directly. While for the other dynamical systems, we construct the temporal networks on the basis of the original phase space points. In fact, the results are unchanged when taking a phase space reconstruction from the *x*-coordinate, see in Fig. 2(c), where we take the Hénon map in the chaotic regime as an example. As shown in Fig. 2(c), profiles of *H* versus *τ* for different time delays *l* are similar to that of the original phase points exhibited in Fig. 2(b). Moreover, we show that the memory exponent is invariant even under a nonlinear transformation of phase space. This is illustrated in Fig. 2(d), where profiles of *H* versus *τ* computed from the same chaotic time series of the Hénon map in Fig. 2(b), however, passed through quadratic and cubic nonlinear transformations of the phase space. The results in Fig. 2(d) are almost identical to that from the original data, presented in Fig. 2(b).

### The memory entropy analysis of bifurcation phenomena

**Figure 3:**
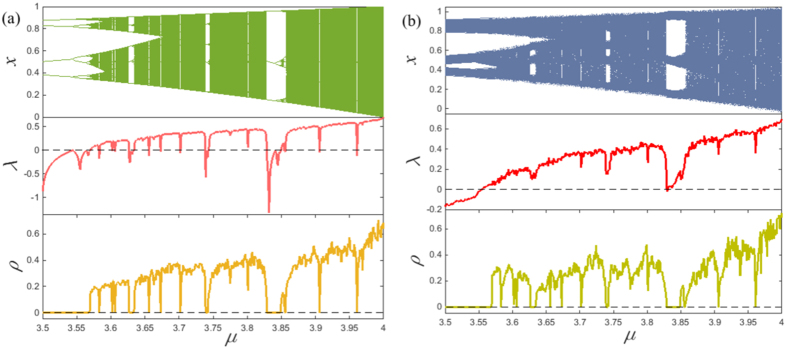
(**a**) The bifurcation diagram, the largest Lyapunov exponent *λ* and the memory exponent *ρ* for the logistic map in the range 3.5 ≤ *μ* ≤ 4. (**b**) The influence of additive Gaussian noise (SNR = 28.5 dB) on the bifurcation diagram, largest Lyapunov exponent *λ* and the memory exponent *ρ*. Note that here for each *μ*, we choose the unified embedding parameters (i.e., *m* = 2 and *l* = 1) for obtaining the quantities *ρ* and *λ* in both situations (i.e., with and without the Gaussian noise).

Furthermore, we show that the memory exponent *ρ* can be used to characterize the bifurcation phenomena of dynamical systems. Here, we select the logistic map (i.e., *x*_*n*+1_ = *μx*_*n*_(1 − *x*_*n*_)) as a benchmark example and select *μ* ∈ [3.5, 4] with step size Δ*μ* = 0.001. For each *μ*, we record 10^4^ time points removing the leading 4000 observations (to eliminate transient states). Since the considered example is a one-dimensional map^33^,^34^, we perform the phase space reconstruction for each recording with the embedding dimension *m* = 2 and the time delay *l* = 1 and execute the ME analysis with *N* = 900. As shown in Fig. 3(a), the memory exponent *ρ* is sensitive to the presence of dynamical transition in the logistic map. The pattern of *ρ* versus *μ* not only correctly locates the periodicity of the transient periodic motions but also successfully reveals the intensity level of the chaotic behavior. In particular, for the chaotic regimes, the trend is very similar to that from the largest Lyapunov exponent *λ* calculated using the TISEAN software package^35^. The similarity is supported by observing the correlation coefficient *r* with *r* = 0.93. Meanwhile, *ρ* = 0 is obtained for the periodic regimes, as expected. These results indicate that the memory exponent *ρ* serves as an effective and alternative metric for characterizing different dynamical regimes. Moreover, we introduce observational noise into the recordings of each *μ* with the medium level (i.e., signal-to-noise ratio (SNR) is 28.5 dB). The periodic windows are then obscured by observing the bifurcation diagram in Fig. 3(b). Nonetheless, we can see that the memory exponent *ρ* can successfully detect periodic windows in the presence of medium level Gaussian noise. However, the traditional largest Lyapunov exponent *λ* fails in the same scenario as *λ* > 0 for the majority of these periodic windows, see in Fig. 3(b). The results show that the memory exponent *ρ* is more robust in comparison with the largest Lyapunov exponent *λ*. Note that here we choose the unified embedding parameters (i.e., *m* = 2 and *l* = 1) for calculating the quantities *ρ* and *λ*.

### The betweenness preference phenomena of chaotic time series

**Figure 4:**
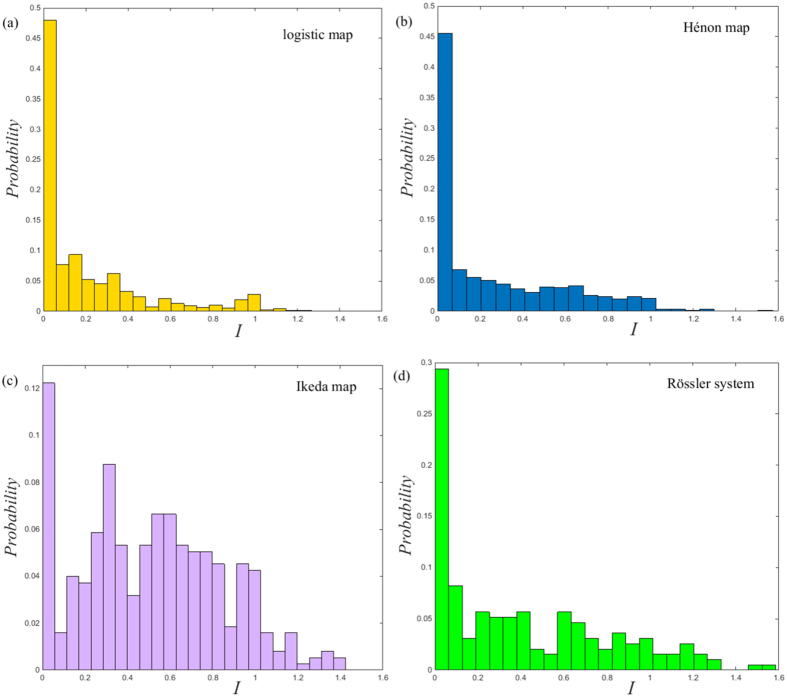
Betweenness preference distributions for the previous temporal networks constructed from (**a**) the logistic map, (**b**) the Hénon Map, (**c**) the Ikeda Map, and (**d**) the Rössler system.

**Figure 5:**
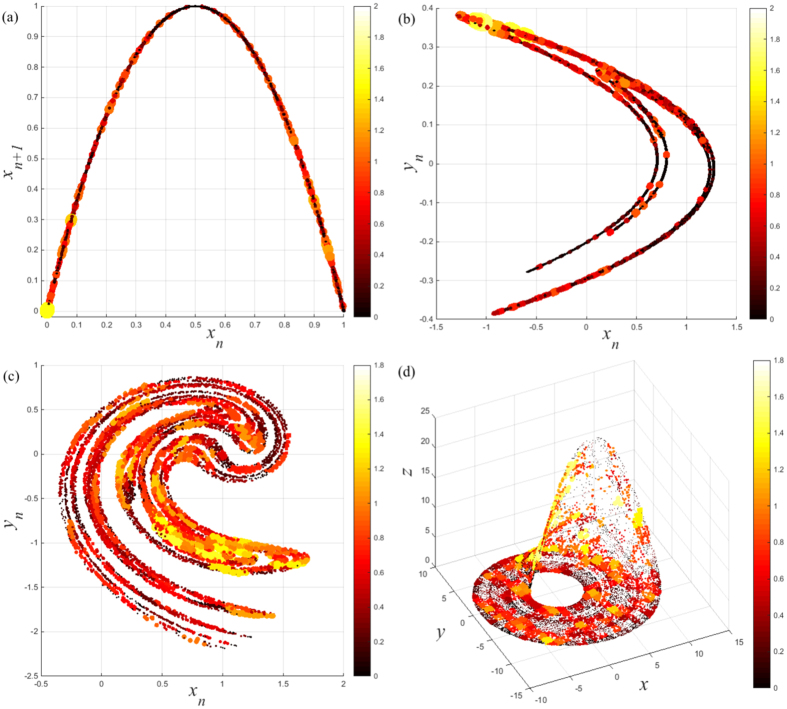
Colour-coded representation of the betweenness preference in phase space of (**a**) the logistic map, (**b**) the Hénon Map, (**c**) the Ikeda Map, and (**d**) the Rössler system.

The previous ME analysis provides only a part of the temporal network perspective of an observed time series. We further employ the betweenness preference to extract more interesting features. The betweenness preference characterizes the preferential connection of nodes in a temporal sense^23^. Specifically, first derive a betweenness preference matrix *B*^*υ*^(*t*) from a constructed temporal network *G*^*T*^, whose entries 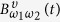 = 1(0) if the path (*ω*_1_, *υ*; *t*) is (not) followed by the path (*υ, ω*_2_; *t* + 1). Second, summarize the 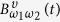 over all *t* to obtain a time-aggregated betweenness preference matrix *B*^*υ*^ such that 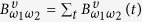. Finally, calculate the betweenness preference measure *I*^*υ*^ for the node *υ* as follows:


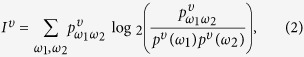


where 
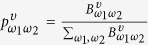
, 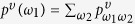, and 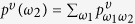. In this context, a trivial result is that *I*^*υ*^ = 0 for the temporal network constructed from periodic time series. In contrast, for chaotic time series, the resultant temporal network presents a pronounced amount of betweenness preference distributions shown in Fig. 4. These interesting distribution patterns are related to the evolution behaviors of distinct dynamical systems. In fact, it is well known that the skeleton of a chaotic attractor is made up of an infinite number of unstable periodic orbits^29^. The trajectory will typically switch or jump among these UPOs with different preferences in a temporal sense, something which is captured well by the betweenness preference analysis. Specifically, for the logistic map and the Hénon Map, the distribution of betweenness preference is heterogeneous with a relatively high probability of *I* = 0. This characteristics implies that the movement of their trajectories has a weak correlation in phase space. In contrast, the betweenness preference distribution for the Ikeda map is rather homogeneous and broad, meaning that the motion of the trajectory among different UPOs is highly correlated. However, none of them matches that of the Rössler system, which lies somewhere between heterogeneous and homogeneous as illustrated in Fig. 4(d). The result hints at the existence of a biased preference for its trajectory evolution in phase space. Moreover, for better illustration, we further reflect the spatial distribution of the betweenness preference shown in Fig. 5. We find high values of *I* near the tips of the attractor in phase space, and low values almost filled the whole phase space regions. Interestingly, there are pronounced regions with rather few isolated points of high *I* in phase space. Clearly, they play an important role in linking the evolution of trajectory among different regions in phase space in a temporal sense. Although we do not provide a complete explanation of these interesting features, the betweenness preference analysis allows to uncover the preference behavior of trajectory evolution of dynamical systems.

### The temporal network perspective of human ECG data

**Figure 6:**
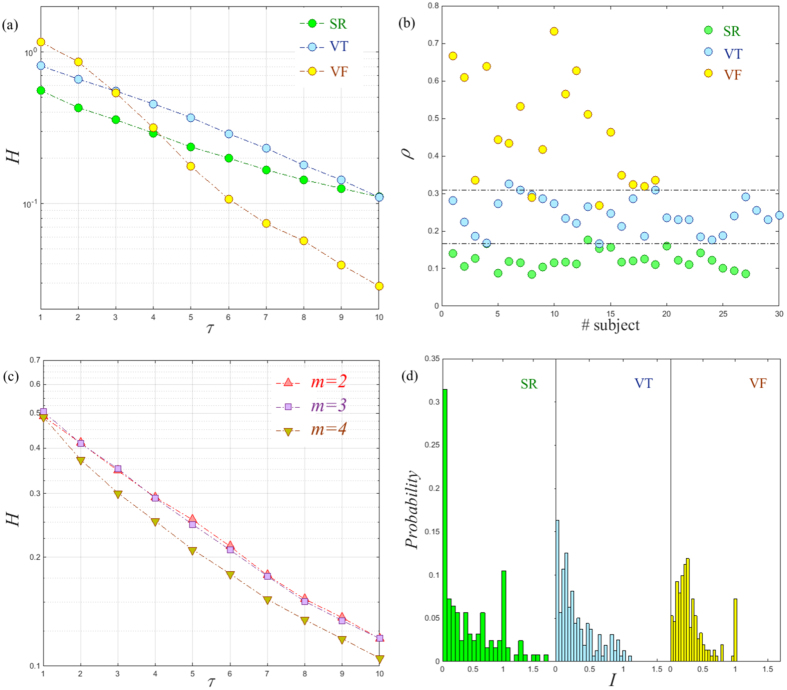
(**a**) The entropy growth rate *H* of *τ*-order aggregate network with the memory scale *τ* in temporal networks generated from the representative SR, VT, and VF recordings. (**b**) Values of the memory exponent *ρ* for the SR, the VT, and the VF groups. Note that here we choose the embedding dimension *m* = 2, the time delay *l* = 15, and the partition *N* = 300 for these physiologic time series. (**c**) The entropy growth rate *H* as a function of *τ* for the representative SR recording with different embedding dimensions *m*. (**d**) Betweenness preference distributions for the representative SR, VT, and VF recordings.

Finally, we consider the application of the temporal network analysis to human ECG to reveal the memory effect and betweenness preference within the human cardiac system. Here, the human ECG recordings are collected from patients in a Coronary Care Unit and adjacent Cardiology Ward containing three different states: SR, VT, and VF, as described in ref. 36. Each recording consists of 10,000 data points (20 s) in length. We repeated the calculation applied to previous time series with these physiological signals. We observe that the entropy growth rates *H* decrease exponentially with memory scale *τ* (see Fig. 6(a) for three representative subjects) consistent with that of chaotic systems. These findings suggest that the human cardiac system has chaotic characteristics, which is consistent with several previous results^5^,^6^. Meanwhile, we note that the slopes of log_10_*H* versus *τ* are different for distinct types of the ECG recordings in Fig. 6(a). This is further supported by observing the memory exponent *ρ* shown in Fig. 6(b), where distinct types of the ECG recording lie in a different range of values *ρ*. Hence the ME analysis uncovers the underlying mechanism (i.e., memory effect) that governs each type of human ECG recording. For example, for the SR group, the cardiac system presents a regular and repeated behavior. Therefore, the memory effect has less influence on the function of the cardiac system which in turn results in a smaller memory exponent *ρ*. In contrast, for the VF group, the cardiac system is under extreme physiological stress and presents more complex irregular behavior^37^. In such case, the memory effect seemingly affects the cardiac system more strongly. Since the condition of VT groups usually lies between regular and extremely disordered coronary rhythm their corresponding memory exponent *ρ* is in the middle as expected. Hence, the ME approach provides a powerful tool in characterizing human cardiac system from another perspective beyond the nonlinear dynamics aspect.

Since the recordings are non-stationary and noisy, we choose the embedding dimension *m* = 2 based on the information theoretic criterion^38^. Although the embedding dimension is substantially smaller than one would reasonably expect, it can be adopted to classify different states using their trajectories^39^. Loosely speaking, embedding is akin to “unfolding” the dynamics, albeit from different axes. Here, we show that the results are unchanged when slightly increasing the embedding dimension. For convenience, we take the representative SR recording as an example. It is shown that profiles of *H* versus *τ* for different embedding dimensions *m* present a similar tendency, seen in Fig. 6(c). Regarding the significance of the ME analysis in characterizing real world data, in the future we can adopt the surrogate method as a hypothesis model^4^. More precisely, first, we use the surrogate method to generate an ensemble of surrogate data from an observed time series with a desired property. We then calculate the memory effect of both the original time series and the surrogates. Finally, we can assess the statistical significance of the ME analysis through surrogate test technique.

The temporal network view of the ECG recordings enhances our understanding of the human cardiac system. Besides the previous memory effect, we further show that the betweenness preference analysis can reveal the significant difference among the SR, VT, and VF recordings. Here, for convenience, we take the results of the representative SR, VT, and VF recordings as an example shown in Fig. 6(d). Clearly, different recordings exhibit remarkably distinct distribution patterns. Specifically, the distribution pattern of the SR recording shows a relative high probability of *I* = 0. However, the probability of the same value is much smaller for that of the VF and VT recordings. Such clear difference is related to the distinct behaviors of the human cardiac system at health and pathological state. In particular, the cardiac system tends to have a weak correlation in the health state. Meanwhile, we notice that for the SR and VF recording, a spike emerges at the position of *I* = 1. However, this remarkable feature is almost absent in that of the VT recording. Although we do not provide a complete explanation to these distinct characteristics, the betweenness preference analysis indeed allows one to classify and characterize the human cardiac system from a new perspective.

## Discussion

In summary, we develop a transformation from time series to temporal networks by unfolding temporal information into an additional topological dimension. For the transformed temporal network, we first introduce the so-called ME analysis to characterize the memory effect within the observed signal. We find that the memory effect can accurately differentiate various types of time series including white noise, 1/f noise, AR model, periodic and chaotic time series. Interestingly, an exponential scaling behavior emerges for a chaotic signal and the memory exponent is in good agreement with the largest Lyapunov exponent. Moreover, such memory exponent is capable of detecting bifurcation phenomena and characterizing real systems, for example, the human ECG recordings from the SR, VT and VF states. Moreover, we further adopt the betweenness preference analysis to characterize and classify dynamical systems and separate the human cardiac system under different states. Our work uncovers the function of the memory effect and betweenness preference in governing various dynamical systems. In the future, a number of other statistics that were recently developed in temporal networks, could be applied after our transformation scheme to shed light on the dynamics of time series.

We notice that the ME analysis is based on entropy rate of the consecutive memory network versus the memory scale, which seems to be related to K2 entropy^40^,^41^ and Markov models^42^. However, these methods approach the problem from different angles and with different intentions. Specifically, for using Markov models to characterize dynamical systems, they usually assume that the current state of a dynamical system provides all the information to predict future states and the history information is redundant. Although they are successfully adopted for prediction, especially to financial time series, the Markov models may fail to capture the high-order correlations underlying dynamical systems. While for the dynamical invariant K2 entropy, it is a classic quantity for quantifying “how” chaotic the signal is refs 40 and 41. On this point, the ME analysis seems to play the same function as that of the K2 entropy. Nonetheless, they have different channels to reveal such interesting metrics. In particular, for the K2 entropy, it is usually obtained by adapting embedding dimensions^40^ or using recurrence plots^41^. In contrast, the ME analysis is calculated in the temporal network paradigm. The advantage of using a temporal network representation is that we can derive a series of high-order memory networks. The properties of these memory networks will benefit us to explore more interesting high-order level features of an observed signal, which are absent from the previous techniques.

Finally, there is a long tradition of using symbolic dynamics to characterize time series, of which the works of refs 43, 44 and 45 are now the classic examples. The motivation for our technique is similar — the dynamics is partitioned and we look for transitions. Moreover, both the ME analysis and the symbolic dynamics are based on the dynamical entropy for deriving the “memory” stored in time series and their results are closely related to the Kolmogorov-Sinai entropy, which can quantify the order and randomness of an observed time series^46^. On this point, we admit that both methods have some similarities but are not identical. In fact, we believe that our temporal network method builds on and develops the traditional idea of symbolic dynamics. Nonetheless, there is a major difference between our method and the symbolic dynamics approach. Specifically, the methods in refs 43, 44 and 45 treat the evolution of dynamical systems using a signal Markov chain. In contrast, the memory effect analysis is based on the entropy rates of consecutive memory networks constructing from the temporal network. Furthermore, as we declared earlier, the other properties of these memory networks will bring new insights into the high-order level features of dynamical systems, which are absent from the symbolic dynamics techniques. Nonetheless, the traditional symbolic dynamics may be of benefit to us when we seek to further develop the theoretical aspect of the ME analysis in the future. Of course, the memory effect is part of our findings in virtue of the temporal network representation. With the help of our unique transformation from time series to temporal networks, we can adopt other statistics and tools in the temporal network regime to reveal more interesting features of time series, for example, the betweenness preference analysis shown in Fig. 5. Moreover, although we have revealed the memory effect of dynamical systems here, the technique (i.e., the ME analysis) presented in this paper should prove useful also for characterizing some real systems describing by temporal networks as well, for example, lottery, traffic system, and football games. This will be another interesting application of our memory effect analysis.

## Methods

### The memory entropy analysis of a temporal network

After deriving the temporal network *G*^*T*^ from an observed time series, we introduce memory entropy to reveal the effect of memory on shaping its dynamics. First, we build a null aggregate network *G*^(0)^ in which a directed edge (*υ, ω*) from node *υ* to *ω* exists whenever a time-stamped edge (*υ, ω*; *t*) emerges in *G*^*T*^ for some *t* and the weight *w(υ, ω*) measures how many times the directed edge (*υ, ω*) occurs in the temporal network *G*^*T*^. Second, we construct the consecutive memory network *G*^(*τ*)^ = (*V*^(*τ*)^, *E*^(*τ*)^), controlled by the memory factor *τ*. Specifically, the node 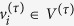 represents a possible *τ*-step paths in the network *G*^(0)^ such that 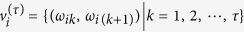, where 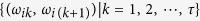 is a set of consecutive edges in *G*^(0)^. Edges *E*^(*τ*)^ in *G*^(*τ*)^ are defined by all possible paths of length 2*τ* + 1 in *G*^(0)^ and we use 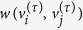 to measure how frequently the *τ*-step paths 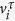 and 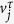 emerge together in *G*^*T*^. More precisely, 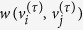 is given by


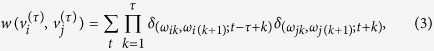


where 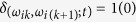 if the time-stamped edge (*ω*_*ik*_, *ω*_*i(k*+1)_; *t*) (not) exists in the temporal network *G*^*T*^. Third, for the memory network *G*^(*τ*)^, we define the transition probability 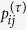 from node 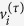 to node 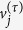 as follows


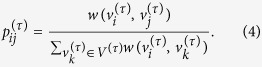


From the above transition probability, we calculate the entropy growth rate *H(τ*)


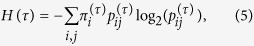


where 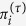 is the *i*th component of the stationary distribution. The entropy growth rate measures the uncertainty of the next step of the jumps given the current state weighted by the stationary distribution^25^,^47^. In this sense, the less effect memory has in the trajectory flow Γ, the more the entropy growth rate will decrease in the high-order memory network *G*^(*τ*)^. Finally, we plot the entropy growth rate *H(τ*) as a function of the memory scale *τ*. We call this the memory entropy analysis of a temporal network.

## Additional Information

**How to cite this article**: Weng, T. *et al*. Memory and betweenness preference in temporal networks induced from time series. *Sci. Rep.* **7**, 41951; doi: 10.1038/srep41951 (2017).

**Publisher's note:** Springer Nature remains neutral with regard to jurisdictional claims in published maps and institutional affiliations.
